# Small Interfering RNA Targeting Mitochondrial Calcium Uniporter Improves Cardiomyocyte Cell Viability in Hypoxia/Reoxygenation Injury by Reducing Calcium Overload

**DOI:** 10.1155/2017/5750897

**Published:** 2017-02-27

**Authors:** Yuriana Oropeza-Almazán, Eduardo Vázquez-Garza, Héctor Chapoy-Villanueva, Guillermo Torre-Amione, Gerardo García-Rivas

**Affiliations:** ^1^Cátedra de Cardiología y Medicina Vascular, Escuela de Medicina, Tecnológico de Monterrey, Monterrey, NL, Mexico; ^2^Centro de Investigación Biomédica, Hospital Zambrano-Hellion, San Pedro Garza-García, NL, Mexico; ^3^Methodist DeBakey Heart and Vascular Center, The Methodist Hospital, Houston, TX, USA

## Abstract

Intracellular Ca^2+^ mishandling is an underlying mechanism in hypoxia/reoxygenation (H/R) injury that results in mitochondrial dysfunction and cardiomyocytes death. These events are mediated by mitochondrial Ca^2+^ (*m*Ca^2+^) overload that is facilitated by the mitochondrial calcium uniporter (MCU) channel. Along this line, we evaluated the effect of siRNA-targeting MCU in cardiomyocytes subjected to H/R injury. First, cardiomyocytes treated with siRNA demonstrated a reduction of MCU expression by 67%, which resulted in significant decrease in mitochondrial Ca^2+^ transport. siRNA treated cardiomyocytes showed decreased mitochondrial permeability pore opening and oxidative stress trigger by Ca^2+^ overload. Furthermore, after H/R injury MCU silencing decreased necrosis and apoptosis levels by 30% and 50%, respectively, and resulted in reduction in caspases 3/7, 9, and 8 activity. Our findings are consistent with previous conclusions that demonstrate that MCU activity is partly responsible for cellular injury induced by H/R and support the concept of utilizing siRNA-targeting MCU as a potential therapeutic strategy.

## 1. Introduction

Coronary heart disease (CHD) is the leading cause of death in industrialized and third-world countries [1]. The effects of CHD are associated with the negative effects of ischemia-reperfusion. Ischemia-reperfusion injury normally arises in patients presenting myocardial infarction with acute ST-segment elevation in which timely and effective myocardial reperfusion is limiting the infarct size and death. However, several events that appear during myocardial reperfusion can induce further cell damage in a phenomenon known as reperfusion injury [2].

Numerous experimental studies have identified some critical factors that act in concert to mediate the unfavorable effects of reperfusion injury. First, intracellular and mitochondrial Ca^2+^ (*m*Ca^2+^) overload are exacerbated during reperfusion due to oxidative stress-induced disruption of the sarcolemma and sarcoplasmic reticulum membranes [3]. Second, mitochondrial reenergization during reoxygenation allows the recovery of the membrane potential that drives the Ca^2+^ uptake into the mitochondria through the mitochondrial Ca^2+^ uniporter (MCU) channel and subsequently induces* m*Ca^2+^ overload [4]. These events result in mitochondrial dysfunction, leading to cardiomyocyte death by the opening of the mitochondrial transition pore (_*m*_PTP) [5]. Accordingly, targeting oxidative stress and/or modulating the activity of MCU by a selective blocker such as Ru_360_ [6, 7] or the inhibition of _*m*_PTP opening by cyclosporine A (CsA) provide specific targets for intervention [8].

The role of MCU in the regulation of* m*Ca^2+^ overload is supported by the observations that, in a model of cardiac ischemia-reperfusion, treatment with Ru_360_ decreased the [Ca^2+^]_m_ and maintained mitochondrial ATP synthesis [6]. Ru_360_-treated hearts following in vivo reperfusion were less prone to undergo mitochondrial permeability transition (_*m*_PTP) and apoptosis [9, 10]. The recent description of the molecular identity of the MCU channel [11, 12] and several subunits that are crucial for* m*Ca^2+^ uptake allowed for the development of adult heart-specific transgenic models [13]. In this regard, conditional cardiac-specific MCU^−/−^ mice subjected to reperfusion resulted in significant reduction of* m*Ca^2+^ overload that disabled the activation of _*m*_PTP and inhibited cardiomyocytes apoptosis and necrosis [14, 15]. These data together provide experimental rationale to develop strategies to target MCU activity with the purpose of preventing ischemic-reoxygenation injury.

On the other hand, small interfering RNA- (siRNA-) based treatments provide a potential therapeutic strategy to block MCU activity. Of interest, several phases I and II clinical studies involving patients with hepatitis, hypercholesterolemia, macular degeneration, and solid tumors have been completed [16] and appear to be safe strategy for gene therapy in humans [17].

Therefore, the purpose of this study was to explore the potential use of siRNA-targeting MCU in an in vitro model of hypoxia/reoxygenation (H/R) injury. Our results confirm previous observations that define the MCU as an essential modulator of the detrimental effects of reperfusion injury and suggest that MCU silencing using siRNA has the therapeutic potential to be used as a cardioprotective strategy.

## 2. Materials and Methods

### 2.1. Reagents

All chemical reagents, cell culture media and supplements, siRNA, and fluorescent probes were purchased from Sigma-Aldrich (St. Louis, MO, USA), unless otherwise stated.

### 2.2. MCU siRNA Sequence

To silence MCU, specific siRNA sequence was designed using RNAi siDirect v2.0 with the lowest predicted off-target potentials and 100% homology with the rat gene (NCBI Reference Sequence NM_001106398.1). Then, we used siRNA-MCU Calcium1, nt 761–779 of the corresponding mRNA, sense strand sequence: 5′-CGGCUUACCUGGUGGGAAU-3′. siRNA duplex was synthesized by Sigma-Aldrich (St. Louis, MO, USA). The nontargeting siRNA sequence is the MISSION® siRNA Universal Negative Control #1 (SIC001) named as siRNA-Neg.

### 2.3. Cell Culture and siRNA Transfection

Rat ventricular myocardial H9c2 cell line (CRL-1446™) was obtained from ATCC® (Manassas, VA, USA). Cells were grown in Dulbecco's modified Eagle's medium (DMEM) (D7777) and supplemented with 10% fetal bovine serum (FBS) Invitrogen (Carlsbad, CA, USA) and 1x penicillin-streptomycin (P4333) in a humidified incubator at 37°C with 5% CO_2_ and 95% air. For MCU silencing, H9c2 cells were seeded at 2.5 × 10^4^ cells per well in 12-well plates and 24 h later were transfected with 18.8, 112.5, or 225 nM of siRNA-MCU designed or siRNA-Neg using the HiPerFect™ Transfection Reagent (Qiagen®, Venlo, Netherlands), according to the manufacturer's protocols.

### 2.4. RNA Extraction, Reverse Transcription, and Gene Expression Analysis by Quantitative PCR

For the gene expression analysis of MCU silencing, after 96 h of transfection, total RNA was isolated from H9c2 cells by homogenization in TriReagent™ following manufacturer's instructions. The RNA quantification and purity assessment was performed with a Take3™ Micro-Volume plate used in the microplate spectrophotometer Synergy™ HT (BioTek® Instruments, Winooski, VT, USA). From one microgram of total RNA of each sample, cDNA was synthesized with the ImProm-II™ Reverse Transcription System (Promega®, Madison, WI, USA) and 5 ng was analyzed by qRT-PCR SensiFast™ SYBR® Lo-Rox Kit (Bioline®, London, UK). The housekeeping gene *β*-actin was used to normalize all data. Real-time PCR primer sequences to amplify a fragment of 158 bp of CCDC109A (MCU) gene are the following: fw, 5′-CACACAGTTTGGCATTTTGG-3′, and rv, 5′-TGTCTCTGGCTTCAGGATAA-3′. The primer sequences to amplify a fragment of 110 bp of *β*-actin are fw, 5′-GAAAAGATGACCCAGATCATG-3′, and rv, 5′-ATCACAATGCCAGTGGTAC-3′. Comparing expression analysis in siRNA-MCU cells to siRNA-Neg cells was performed using 2^−ΔΔCt^ method.

### 2.5. Western Blot Assay

Cell proteins were extracted after 96 h of H9c2 transfection, as previously described [18]. Briefly, after being washed with cold PBS, cell cultures were scraped, harvested, and resuspended in RIPA buffer. The samples were vortexed and sonicated on rounds of 10 sec sonication/10 sec rest and centrifuged at 15000 ×g 10 min. The protein concentration of the lysate was determined by Lowry protein assay. Protein lysates (30 *μ*g/lane) were resolved on SDS-PAGE gel 10%, transferred onto PVDF membrane at 150 mA, 50 min, and incubated with anti-MCU antibody ab121499 (Abcam, Cambridge, MA, USA) 1 : 500 and washed three times for 10 min with PBS-Tw 0.5% and subsequently probed with secondary antibody anti-rabbit IgG conjugated with HRP (Millipore, Billerica, MA, USA) 1 : 5000 for 2 h at room temperature (RT). After washing three times for 10 min, protein-antibody blots were developed with Clarity™ Western ECL (Bio-Rad, Hercules, CA) and quantified by using a BioSpectrum 415 Image Acquisition System (UVP®, Upland, CA, USA). Anti-*β*-actin antibody (ab8229) 1 : 500 or anti-GAPDH antibody (ab9484) 1 : 500 (both of them from Abcam, Cambridge, MA, USA) was used as a loading control. The level of MCU expression was the ratio of intensities of the MCU-signal/*β*-actin signal normalized versus siRNA-Neg signal.

### 2.6. Measurement of the Mitochondrial Ca^2+^ Uptake

Cytosolic-free Ca^2+^ was monitored in digitonin-permeabilized H9c2 transfected cells. Briefly, 5 × 10^4^ cells were washed three times in Tyrode solution (in mM: 128 NaCl, 0.4 NaH_2_PO_4_, 5 glucose, 5.4 KCl, 0.5 MgCl-6H_2_O, and 25 HEPES, pH 7.4) without Ca^2+^ and resuspended in 50 *μ*L of respiration buffer containing in mM the following: 150 sucrose, 50 KCl, 2 KH_2_PO_4_, 20 Tris-HCl pH 7.3, 5 succinate, 2 *μ*g/mL rotenone, 40 *μ*M digitonin, 0.5 *μ*M thapsigargin (TG), 1 *μ*M CsA, 0.3 *μ*M Calcium Green-5N (CG-5N) salt-free (Thermo Fisher Scientific, Waltham, MA, USA). CG-5N fluorescence (*λ*_ex_485 nm/*λ*_em_528 nm) was monitored at 25°C at basal conditions and after 15 *μ*M Ca^2+^ addition with constant agitation using a microplate fluorescence spectrophotometer Synergy HT (BioTek Instruments, Winooski, VT, USA).

### 2.7. Mitochondrial Membrane Potential (Δ*ψ*_*m*_) and _*m*_PTP Measurements

Safranin was used to assess Δ*ψ*_*m*_ in digitonin-permeabilized transfected cardiomyocytes as previously described by Oliveira et al. [19], with some modifications. Briefly, 5 × 10^4^ H9c2 transfected cells were washed and resuspended as already stated in 50 *μ*L of respiration media with 2 *μ*M safranin. Safranin fluorescence was recorded at *λ*_ex_485 nm and *λ*_em_590 nm using a microplate fluorescence reader. After 5 min, 10 mM succinate was added as substrate until steady state was reached. A Ca^2+^ pulse of 7.5 or 15 *μ*M was added to induce permeability transition, and 1 *μ*M cyclosporin A (CsA) or 0.5 *μ*M Ru_360_ was used as pharmacological inhibitors of _*m*_PTP opening and MCU, respectively. Cyanide* m*-chlorophenyl hydrazone (CCCP, 20 *μ*M) was added when indicated to dissipate Δ*ψ*_*m*_. Depolarization was measured after 13 min of Ca^2+^ addition. Experimental data were normalized to the maximum safranin fluorescence (100%  Δ*ψ*), calculated as the difference between the steady state fluorescence and after addition of CCCP.

To support that Ca^2+^ induces _*m*_PTP opening, with 5 × 10^4^ H9c2 permeabilized transfected cardiomyocytes in respiration media, we carried out a Ca^2+^ retention experiment with a single 50 *μ*M Ca^2+^ bolus using CG-5N as a Ca^2+^ indicator. The experiment was performed with (1 *μ*M) or without CsA.

### 2.8. Flow Cytometry Measurements

Transfected cardiomyocytes were stained and analyzed in a FACSCanto II™ Flow Cytometer (BD Bioscience, San Jose, CA), and each time point analysis consisted of 5,000 recorded events. Cell morphology was assessed by analyzing the Forward Scatter (FSC) and Side Scatter (SSC) parameter and we excluded doublets by FSC-A/FSC-W gating. Debris was discarded by its distinct low Forward and Side Scatter. Samples were run uncompensated and the FMI analysis was performed using the FlowJo™ vX.07 (Tree Star, Ashland, OR, USA).

#### 2.8.1. Measurements of ROS Production

To assess mitochondrial ROS (superoxide) production, transfected cardiomyocytes were stained with MitoSOX Red (Thermo Fisher Scientific), as previously reported by Mukhopadhyay et al. [20]. Briefly, cells were allowed to load MitoSOX for 30 min at 37°C. After staining, the cells were washed with Tyrode solution. Finally, each sample was stimulated with TG at a concentration of 5 *μ*M. Before TG addition, siRNA-Neg cardiomyocytes were incubated with or without 5 *μ*M Ru_360_ for 30 min or 1 *μ*M CsA during 10 min as pharmacological inhibitors of superoxide production. Serial analysis of the samples was performed at different time points (0.5, 15, 30, 60, and 120 min). The data of the MitoSOX are presented as the fold increase of median intensity fluorescence against the siRNA-Neg controls.

#### 2.8.2. Cytotoxicity Assay

Cell viability and apoptotic and necrotic cell death were measured by Annexin V/PI staining followed by flow cytometry analysis. Briefly, after normoxy (Nxy), hypoxia, and 1.5 and 3 h reoxygenation conditions, H9c2 transfected cells were washed, resuspended in 195 *μ*L Tyrode + 2.5 mM CaCl_2_ solution, and stained with 5 *μ*L of Annexin V Apoptosis Detection Set PE-Cy7 88-8103 (eBioscience, San Diego, CA, USA) for 10 min. Later, cells were washed and resuspended in 195 *μ*L Tyrode + 2.5 mM CaCl_2_ solution and incubated with 100 ng of PI (P4170) to discard necrotic cells. Stained cells were immediately analyzed by flow cytometry. The assay was performed with a two-color-analysis of PE-Cy7-labeled Annexin V binding and PI dye excited with 488 nm laser. To evaluate the proportions of viable, apoptotic, and necrotic cells, we performed fluorescence compensation. After discarding doublets, the living cells (Annexin V^−^/PI^−^, Q4), early apoptotic cells (Annexin V^+^/PI^−^, Q3), late apoptotic cells (Annexin V^+^/PI^+^, Q2), and necrotic cells (Annexin V^−^/PI^+^, Q1) were distinguished.

### 2.9. Measurement of ATP Content

Intracellular ATP content was measured in 96 h transfected cardiomyocytes, using the CellTiter-Glo® Luminescent Assay (Promega, Madison, USA), according to the manufacturer's protocol. The ATP content is expressed as luminescence relative units (LRU).

### 2.10. In Vitro Hypoxia/Reoxygenation Model

**Figure 1:**
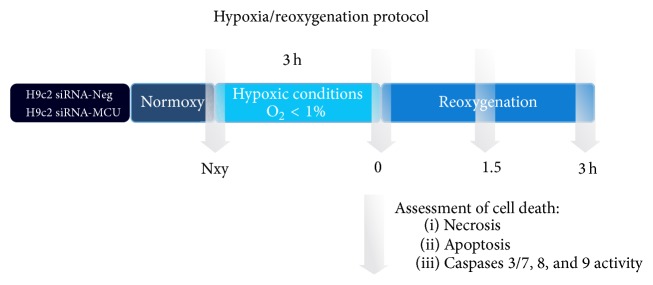
Schematic representation showing the hypoxia/reoxygenation (H/R) protocol applied to each experimental group. At normoxic conditions, siRNA-Neg (control group) and siRNA-MCU cardiomyocytes were subjected to 3 h of normothermic hypoxia (H) with an ischemic Tyrode solution (IT) followed by 3 h of reoxygenation (R). Transfected cardiomyocytes were harvested when indicated by arrows: measurements of necrosis and apoptosis by flow cytometry were performed and caspases 3 and 7 and caspases 9 and 8 activity were determined as described in Materials and Methods. Nxy, normoxy.

For hypoxic challenges, H9c2 transfected cells were trypsinized, washed with Tyrode without glucose, and incubated with a modified Tyrode solution simulating ischemic conditions (IT) (in mM: 135 NaCl, 8 KCl, 0.5 MgCl_2_, 0.33 NaH_2_PO_4_, 5 HEPES, 1.8 CaCl_2_, and 20 Na^+^-lactate, pH 6.8) [21] and transferred into an anaerobic chamber with an oxygen level <1% at 37°C. After 3 h hypoxia, cells were washed and incubated with Tyrode (plus 5 mM glucose, 1.8 mM CaCl_2_) and transferred into an incubator in normoxic conditions (37°C with 5% CO_2_ and 95% air) for 1.5 and 3 h for reoxygenation and analyzed for apoptosis and necrosis at each time. During hypoxia, an experimental group remained in normoxic conditions. A schematic representation of hypoxia/reoxygenation model is shown in Figure 1.

### 2.11. Caspase Activity Measurements

For caspase activity measurements, H9c2 were seeded at 1.5 × 10^3^ cells per well in 96-well plates and 24 h later were transfected as described previously. After 96 h of transfection, the cells were washed with Tyrode, incubated with IT solution, and transferred into an anaerobic chamber with an oxygen level <1% at 37°C. After 3 h hypoxia, IT solution was removed and cells were incubated with DMEM at normoxic conditions (37°C with 5% CO_2_ and 95% air) for 0, 1.5, and 3 h reoxygenation and analyzed for caspases 3 and 7 and caspases 8 and 9 activity at each time. During hypoxia, an experimental group remained in normoxic conditions. The activity of caspases 3 and 7 and caspase 9 and caspase 8 was measured using Caspase-Glo 3/7, 9, and 8 assay (Promega, Madison, WI, USA), respectively, according to the manufacturer's protocols.

### 2.12. Statistical Analysis

Statistical data are presented as mean ± SEM. Comparisons between means were made by unpaired Student's *t*-test or one-way ANOVA followed by Dunnett's, Tukey's, or Bonferroni's post hoc tests when appropriate to compare experimental groups. Differences were considered significant when *P* < 0.05. Data processing, graphs, and statistical analysis were performed with GraphPad Prism (V.5.01, La Jolla, CA, USA) and OriginPro 8.1 SR3 v8.1 (OriginLab Corporation, Northampton, MA, USA).

## 3. Results

### 3.1. siRNA-Targeting MCU Efficiently Decreases Mitochondrial Ca^2+^ Transport in Cardiomyocytes

**Figure 2:**
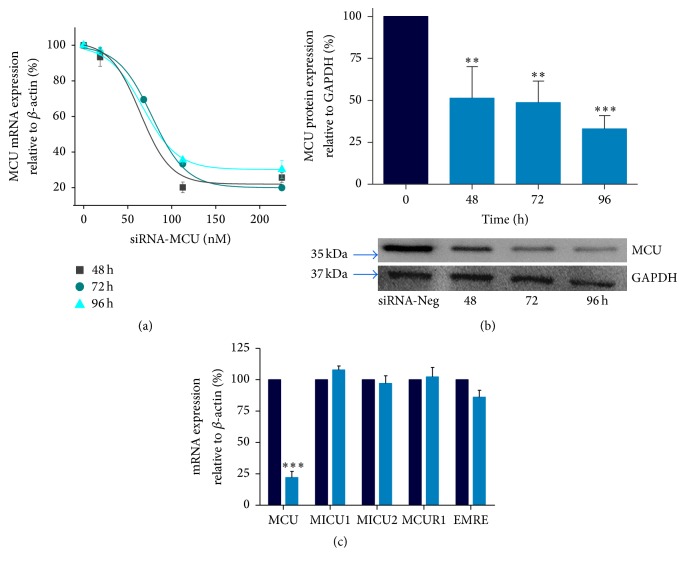
Dose-response analysis of MCU expression in cardiomyocytes transfected with a specific siRNA designed targeting MCU (siRNA-MCU). (a) MCU mRNA expression by qRT-PCR normalized versus *β*-actin using 18.7, 112.5, or 225 nM of siRNA at 0, 48, 72, and 96 h of transfection. We obtained EC50 ∼67.2 nM at 96 h of transfection, mean ± SEM, *n* = 3. (b) Representative protein expression analysis of MCU in cardiomyocytes silenced with 225 nM of siRNA-MCU at 48 h (^*∗∗*^*P* < 0.01); at 72 h (^*∗∗*^*P* < 0.01); and at 96 h of transfection. GAPDH served as a loading control (^*∗∗∗*^*P* < 0.001), mean ± SEM, *n* = 3–6. (c) Relative mRNA abundance of associated regulatory genes in MCU-silenced cardiomyocytes using 225 nM of siRNA-MCU at 96 h of transfection, mean ± SEM, *n* = 4. MICU1, mitochondrial calcium uptake 1; MICU2, mitochondrial calcium uptake 2; MCUR1, mitochondrial calcium uniporter regulator 1; EMRE, essential mitochondrial calcium uniporter regulator.

**Figure 3:**
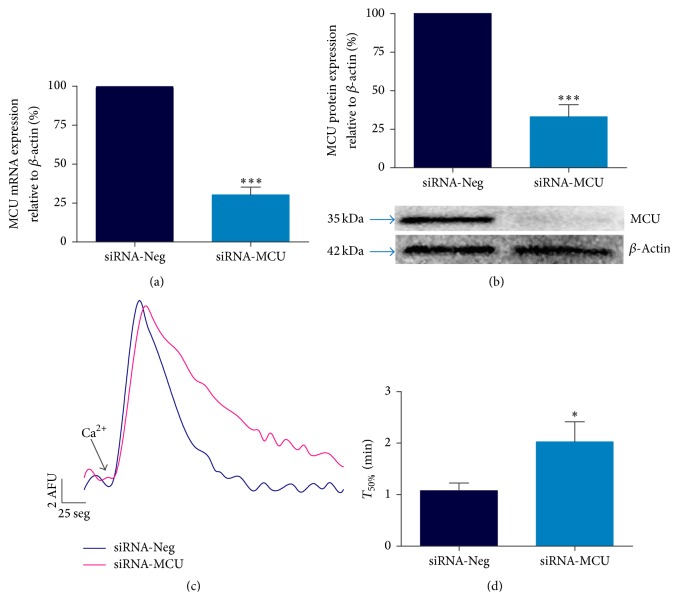
MCU silencing reduces mitochondrial Ca^2+^ transport. (a) MCU mRNA expression by semiquantitative qRT-PCR in silenced cardiomyocytes, ^*∗∗∗*^*P* < 0.001 versus siRNA-Neg, mean ± SEM, *n* = 7. (b) MCU protein expression, with *β*-actin used as a loading control. ^*∗∗∗*^*P* < 0.001 versus siRNA-Neg, mean ± SEM, *n* = 6. (c) Representative traces of mitochondrial Ca^2+^ uptake in permeabilized MCU-silenced cardiomyocytes using CG-5N Ca^2+^ indicator after 15 *μ*M Ca^2+^ addition. The experiment was performed in the presence of 1 *μ*M CsA. AFU, arbitrary fluorescence units. (d) Time to 50% decay analysis (*T*_50%_) of mitochondrial Ca^2+^ transport. ^*∗*^*P* < 0.05 versus siRNA-Neg, mean ± SEM, *n* = 6. MCU silencing conditions were using 225 nM of siRNA-MCU at 96 h of transfection.

Rat cardiomyocytes were transfected with specific MCU-targeted siRNA (siRNA-MCU) or nonsilencing siRNA (siRNA-Neg) to investigate the role of MCU in cell death. Using qPCR assays, we determined that there was a 70% ± 5 (*P* < 0.001) reduction in MCU mRNA expression levels up to 96 h after transfection (Figure 2(a)). Next, our silencing effects were verified using western blot assays. In cardiomyocytes transfected with the siRNA-MCU, MCU expression was lower than that in those transfected with siRNA-Neg cells. This effect showed a time-dependent response with a ~50% silencing at 48 and 72 h (Figure 2(b)). Moreover, at 96 h of transfection, we found significantly lower MCU protein levels by 67% ± 8 (*P* < 0.001). In addition, under this condition the concentration of MCU siRNAs used (225 nM) did not adversely affect the number of viable cells or the cardiomyocyte morphology and ATP production (Suppl. Figure 1A–E, Supplementary Material available online at https://doi.org/10.1155/2017/5750897). Based on these results, we used siRNAs at 225 nM for 96 hrs in the remainder of our experiments, in which we obtained the maximal silencing of MCU mRNA and protein expression (Figures 3(a) and 3(b)). In addition, siRNA-MCU did not modify the relative expression of other uniplex components, such as MICU1, MICU2, EMRE, and MCUR1 (Figure 2(c)).

Figure 3(c) shows representative* m*Ca^2+^ uptake traces for the control and MCU-silenced cardiomyocytes. Permeabilized transfected cardiomyocytes were challenged with a 15 *μ*M Ca^2+^ addition in the presence of 1 *μ*M CsA to determine the maximum MCU activity [9]. In control cells, a rapid* m*Ca^2+^ entry was observed (completely abolished by Ru_360,_ data not shown); however, in siRNA-MCU treated cells, the Ca^2+^ transport was drastically reduced. Mitochondrial Ca^2+^ uptake time to its 50% (*T*_50%_) provides a quantitative index of MCU activity. For the control cardiomyocytes,* T*_50%_ was 1.08 ± 0.22 min, while, in MCU-silenced cells, it was 2.02 ± 0.4 min (*P* < 0.05) (Figure 3(d)). These results indicate a significant reduction in the rate of mitochondrial transport in our MCU-silenced cardiomyocytes.

### 3.2. MCU Silencing Reduces Mitochondrial Ca^2+^ Overload, Permeability Transition Pore, and Oxidative Stress in Cardiomyocytes

**Figure 4:**
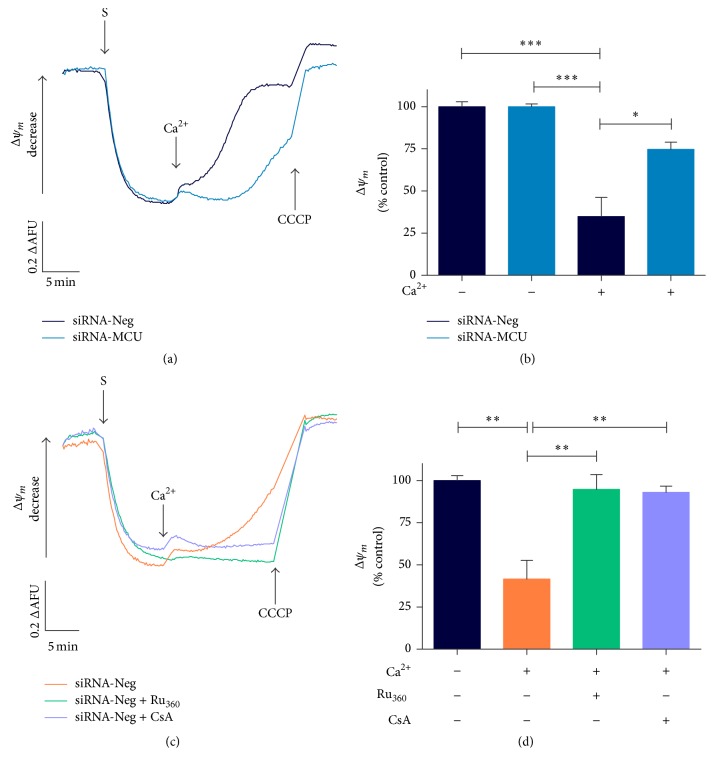
MCU silencing reduces significantly mitochondrial permeability transition by calcium overload. (a) Representative mitochondrial Δ*ψ*_*m*_ traces in permeabilized MCU-silenced cardiomyocytes using 2 *μ*M safranin, after 7.5 *μ*M Ca^2+^ addition. siRNA-Neg cardiomyocytes show no difference in Δ*ψ*_*m*_ compared to MCU-silenced cells at baseline. MCU-silenced cardiomyocytes were able to maintain significantly Δ*ψ*_*m*_ after 7.5 *μ*M Ca^2+^ addition. Upon completion of the traces, 20 *μ*M CCCP was added as uncoupling. (b) Semiquantitative analysis of Δ*ψ*_*m*_ after Ca^2+^ addition in transfected cardiomyocytes. ^*∗*^*P* < 0.05 versus siRNA-Neg, mean ± SEM, *n* = 3. (c) Representative mitochondrial safranin Δ*ψ* recordings in siRNA-Neg cardiomyocytes treated with 5 *μ*M Ru_360_ or 1 *μ*M CsA before 7.5 *μ*M Ca^2+^ pulse. (d) Semiquantitative analysis of Δ*ψ*_*m*_ in siRNA-Neg cardiomyocytes treated with Ru_360_ or CsA. ^*∗∗*^*P* < 0.01 versus siRNA-Neg, mean ± SEM, *n* = 5-4. Upon completion of the registers, 20 *μ*M CCCP was added as uncoupling. All semiquantitative data is normalized to 100% of untreated siRNA-Neg cardiomyocytes at basal conditions. AFU, arbitrary fluorescence units. ^*∗∗∗*^*P* < 0.001 versus siRNA-Neg.

Since excessive Ca^2+^ accumulation in the mitochondrial matrix leads to the collapse of the mitochondrial potential, transfected cardiomyocytes were challenged with Ca^2+^ overload following their effects on the membrane potential. At a steady state, the membrane potential did not show a difference in Δ*ψ*_*m*_ between the control and MCU-silenced cardiomyocytes (Suppl. Figure 1C-D). Nevertheless, after a Ca^2+^ pulse, the control cardiomyocytes showed a significant Ca^2+^-dependent depolarization compared with MCU-silenced cardiomyocytes (Figure 4(a)). At the same time, MCU-silenced cells were able to maintain Δ*ψ*_*m*_ in ~75% (*P* < 0.05) (Figure 4(b)). Ru_360_ and CsA treatment protected against Ca^2+^-induced membrane depolarization by ~95% (Figures 4(c) and 4(d) and Suppl. Figure 2A).

Moreover, in Ca^2+^ retention experiments with a 50 *μ*M Ca^2+^ bolus, we observed an early and dramatic release of Ca^2+^ from control cardiomyocytes compared with the MCU-silenced cells (Suppl. Figure 2B). Ca^2+^ release was inhibited by CsA, indicating the involvement of _*m*_PTP as a trigger of membrane potential collapse.

**Figure 5:**
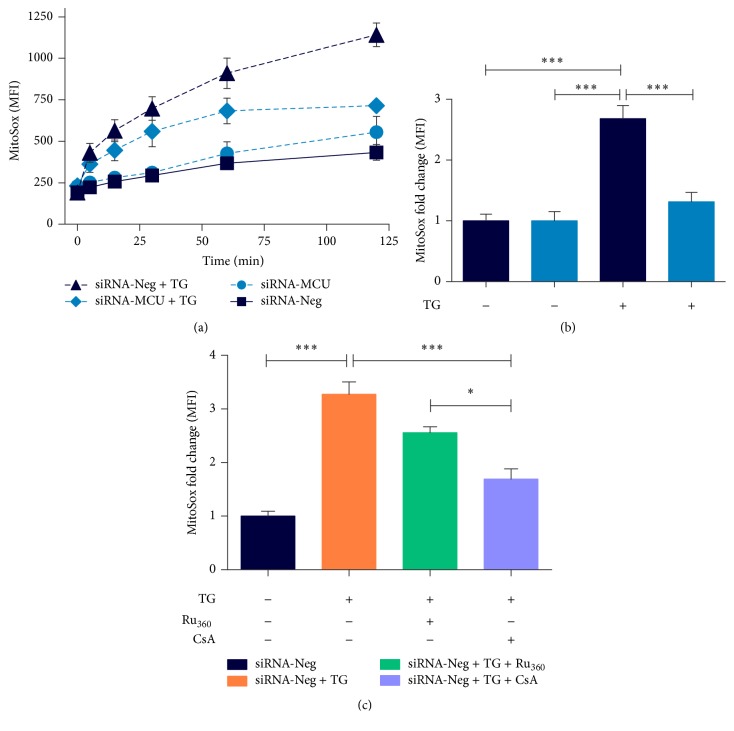
MCU silencing reduces mitochondrial oxidative stress. (a) Time-response curve of mitochondrial ROS (superoxide) production evoked by cytosolic Ca^2+^ overload with 5 *μ*M TG in transfected cardiomyocytes. Measurements were realized over 120 min of TG treatment and ROS was detected with MitoSOX Red by flow cytometry. (b) ROS levels at 120 min of TG addition is presented as FMI fold change with respect to TG untreated cardiomyocytes. ROS production in siRNA-Neg cardiomyocytes was twofold greater than that in MCU-silenced cardiomyocytes after TG addition, ^*∗∗∗*^*P* < 0.001, mean ± SEM, *n* = 5. (c) ROS production in siRNA-Neg cardiomyocytes pretreated with 5 *μ*M Ru_360_ or 1 *μ*M CsA, measured after 120 min of 5 *μ*M TG addition. Data are presented as described previously. ^*∗*^*P* < 0.05 and ^*∗∗∗*^*P* < 0.001; mean ± SEM and *n* = 6–8.

Oxidative stress and Ca^2+^ overload are known to have detrimental effects on mitochondrial membrane integrity since both are inducers of _*m*_PTP opening [22].  Figure 5(a) shows the time-course analysis of superoxide production in response to TG-induced Ca^2+^ overload. At basal conditions, the control and MCU-silenced cardiomyocytes showed a similar fluorescence intensity of MitoSOX. After 60 and 120 min of TG treatment, MitoSOX intensity was significantly higher in siRNA-Neg transfected cells than in MCU-deficient cardiomyocytes (Figure 5(a)). Therefore, MCU-deficient cardiomyocytes showed a 51% (*P* < 0.001) reduction in Ca^2+^ overload-induced superoxide production (Figure 5(b)). Under these conditions, the Ru_360_ and CsA treatment also decreases ROS production by 22% (ns) and 49% (*P* < 0.001), respectively (Figure 5(c)). In accordance with these findings, MCU silencing might exert protective effects by reducing oxidative stress induced by Ca^2+^ overload.

### 3.3. MCU Silencing Reduces Cardiomyocyte Cell Death in Hypoxia/Reoxygenation Injury

**Figure 6:**
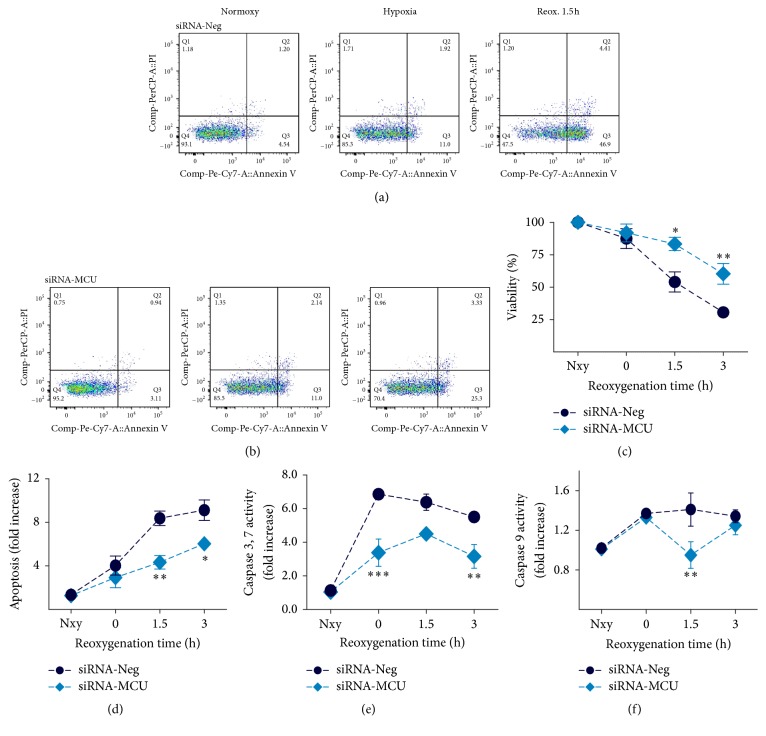
MCU silencing markedly reduced cardiomyocyte cell death in hypoxia/reoxygenation injury. (a and b) Representative dot-plot diagrams of flow cytometry viability/apoptosis analysis of siRNA-Neg and MCU-silenced cardiomyocytes in Nxy, after hypoxia and 1.5 h reoxygenation time. Viability and apoptosis were determined by PI and Annexin V PE-Cy7 conjugated staining, respectively. Q4 quadrant represents the viable cells, Q2 and Q3 represent the apoptotic cells, while Q1 and Q2 are the necrotic population. (c and d) Reoxygenation-time dependent cardiomyocyte viability and apoptosis at 0, 1.5, and 3 h reoxygenation, respectively. ^*∗∗*^*P* < 0.01; mean ± SEM; *n* = 7. Data is presented as percentage of Nxy conditions. (d) Apoptosis was ∼twofold and 1.5-fold increase in siRNA-Neg cardiomyocytes with respect to MCU-silenced cells at 1.5 and 3 h reoxygenation, respectively. Apoptosis is normalized to Nxy and expressed as fold change. ^*∗*^*P* < 0.05, ^*∗∗*^*P* < 0.01, versus siRNA-Neg cardiomyocytes, mean ± SEM, *n* = 7. (e) Time-dependent activation of caspases 3 and 7 and (f) caspase 9 during reoxygenation. ^*∗∗*^*P* < 0.01, ^*∗∗∗*^*P* < 0.001, versus siRNA-Neg cells, mean ± SEM, and *n* = 7; Nxy, normoxic conditions.

Transfected cardiomyocytes were incubated for 3 h in a hypoxic chamber for O_2_ (1%) in combination with glucose-deprivation, serum-free, and acidosis (pH 6.8) conditions to induce hypoxia. A schematic model of hypoxia/reoxygenation is shown in Figure 1. Cell viability and apoptosis were determined by Annexin V/PI staining and analyzed by flow cytometry at specific time points during Nxy and during reoxygenation: at the beginning (0 h), after 1.5 h, and after 3 h. Representative flow cytometry dot plots showing the populations of viable (Annexin V^−^, IP^−^) and apoptotic cells (Annexin V^+^/IP^−^ and Annexin V^+^/IP^+^) are shown in Figures 6(a) and 6(b) at Nxy and 1.5 h of reoxygenation. Cell viability was ~90% (Suppl. Figure 1A) and apoptosis was ~3–5% in both transfected cardiomyocytes at normoxic conditions. At the onset of reoxygenation, we did not find any changes in cell death between the controls (12.6%) and the MCU-silenced cardiomyocytes (8.2%), as shown in Figure 6(c). However, after 1.5 h of reoxygenation, necrosis increased by 46% in the control cardiomyocytes compared to the MCU-silenced cardiomyocytes (16.7%, *P* < 0.05). After 3 h of reoxygenation, the viable cells were preserved in MCU-silenced cardiomyocytes in 60.3 ± 8% and then dropped by 30.5 ± 3.6% (*P* < 0.01) in the control cardiomyocytes. Apoptotic cell death measurements showed similar results; no significant changes were observed in the level of apoptosis at Nxy or the beginning of reoxygenation between the experimental groups. Nevertheless, we found a twofold decrease in MCU-deficient (4.3 ± 1.1) versus siRNA-Neg cardiomyocytes (8.4 ± 0.67) after 1.5 h of reoxygenation (*P* < 0.01). In the same way, after 3 h of reoxygenation, the apoptosis level increased 1.5-fold in siRNA-Neg cardiomyocytes (9.12 ± 0.94 versus 6.04 ± 0.5, *P* < 0.05). Accordingly, caspases 3 and 7 activities increased by 2-fold at the onset of reoxygenation in the control cardiomyocytes (6.86 ± 0.18) compared to the MCU-deficient cardiomyocytes (3.38 ± 0.7, *P* < 0.001). At the same time, caspase 9 activity increased significantly (*P* < 0.001) in both experimental groups, but, after 1.5 h of reoxygenation, the activation levels of caspase 9 dropped 1.5-fold in the MCU-deficient cardiomyocytes (*P* < 0.01). In fact, the activation of caspase 9 and caspases 3 and 7 persisted until 3 h after reoxygenation in siRNA-Neg cells compared to the MCU-silenced cardiomyocytes. Additionally, we observed an 18-fold increase in activation of caspase 8 at the onset of reoxygenation in control cells. Nevertheless, we observed a 40% reduction in caspase 8 activity in MCU-silenced cells compared with control cells (4.95 ± 0.7 versus 8.36 ± 1.6, RLU·mg^−1^). Overall, these results demonstrate that MCU silencing in cardiomyocytes reduces necrosis and apoptosis of the hypoxia/reoxygenation injury.

## 4. Discussion

In the heart,* m*Ca^2+^ uptake can shape cytosolic calcium signals to regulate some physiological processes, principally matching workload and energy production [23]. Nevertheless, an excessive uptake of Ca^2+^ triggers mitochondrial dysfunction through _*m*_PTP opening, leading to cell injury by apoptosis and necrosis [5]. In this regard, several studies have suggested that dysregulation of cytosolic Ca^2+^ which leads to* m*Ca^2+^ overload is present in the physiopathology of ischemia/reperfusion (I/R) injury and heart failure (HF) [24]. The MCU channel, the principal* m*Ca^2+^ transport system, carries out this mechanism. However, the precise role in the regulation of Ca^2+^ handling and its consequences on heart disease remain controversial [25]. Not until recently has the molecular characterization of the MCU been made possible [11, 12], allowing for the study of its univocal role in the physiopathology of myocardial diseases. This finding led us to explore the possibility of modulating MCU expression in knockout mice or using a siRNA technology. In this regard, we tested a specific siRNA design to knock down MCU expression in the cardiomyocyte line for an in vitro MCU target validation. After being challenged to a* m*Ca^2+^ overload, MCU-deficient cells were able to largely sustain Δ*ψ*_*m*_ and retain Ca^2+^ similarly to those pharmacologically treated with CsA, as expected. Altogether, these findings indicate that MCU silencing confers resistance to _*m*_PTP opening [6, 14]. Furthermore, MCU-silenced cardiomyocytes treated with TG (a SR-Ca^2+^/ATPase (SERCA) inhibitor), which promotes a burst of cytosolic Ca^2+^ and later ROS, failed to increase superoxide radical production. Also, after H/R protocol, siRNA-MCU-silenced cardiomyocytes reduced _*m*_PTP opening and activation of caspases 8 and 9. In accordance with our results, caspase 8 might initiate the execution phase of cell apoptosis, after which mitochondrial dysfunction induces activation of caspase 9. One potential explanation for this finding is that caspase 8 activation cleaves Bid [26], and then the cleaved Bid activates mitochondrial Bax/Bak and contributes to multiple mitochondrial dysfunction, including the release of the intramembrane space proteins, and activation of apaf-1, which in turn cleaves the proenzyme of caspase 9 into the active form. These observations are similar to those reported when Ru_360_-MCU was inhibited in in vivo and ex vivo models of I/R [9, 10], confirming that partial or acute MCU inhibition could be a therapeutic strategy to reduce cell death [27]. Moreover, this cardioprotective effect has been linked to MCU as an ischemic preconditioning (IPC) mediator [6, 28]. The mechanisms implicated in IPC are not fully understood; however, low levels of* m*Ca^2+^-generated ROS are involved as a trigger of IPC by activation of survival kinases [29]. Since a large production of ROS during reperfusion contributes importantly to _*m*_PTP opening inducing cardiomyocyte death, the controlled ROS production by MCU silencing mimics the IPC cardioprotective effects. Therefore, our findings are in accordance with the observed protection in conditional cardiac-specific MCU^−/−^ mice [14, 15]. In this transgenic model, deletion of MCU in adult cardiomyocytes leads to protection from cell death induced by acute damage such as ischemia-reperfusion injury. However, the results in MCU-knockout constitutive animals are still controversial. For instance, during reperfusion injury, MCU-knockout hearts were similar to control hearts, and CsA did not exert a protective effect. This suggests that Ca^2+^-independent death pathways take place in the absence of MCU and that alternative mechanisms exist for Ca^2+^ entry [30]. Of note, inbred MCU-knockout mice are not viable because they are embryonically lethal; only mixed-strain MCU-knockout mice are viable [31]. In addition to this controversy, recent results in MCU overexpressing mice are more resistant to reperfusion injury in part due to an enhanced activity of survival kinases [32].

In addition to defining the contribution of MCU activity in pathologies, the discovery of therapeutic strategies that modulate MCU activity will be extremely important for the future development of putative MCU-targeting therapies. Here, using siRNA or several pharmacological strategies (such as Ru_360_ or CsA), we attempted to diminish _*m*_PTP opening or reduce* m*Ca^2+^ overload in order to decrease the likelihood of arrhythmias and postmyocardial infarction cardiac dysfunction [2]. In this regard, in vitro and ex vivo studies of cytosolic Ca^2+^ overload and IR injury have shown that CsA is a potent inhibitor of _*m*_PTP, protecting against reperfusion cardiomyocyte death [8, 33]. Indeed, CsA application in small animal models at the onset of reperfusion has demonstrated a reduction of the myocardial infarct area by ~45% [34] by suppressing Δ*ψ*_*m*_ loss, NADH oxidation, Ca^2+^ release, and mitochondrial swelling [35]. Accordingly, some clinical trials have been performed to determine its protective effect from reperfusion injury in patients with myocardial infarction [36]. Nevertheless, a recent meta-analysis of randomized controlled trials reveals that CsA could not have protective effects towards reperfusion injury in clinical patients [37]. The cause of these effects is not yet clear, but it could be because CsA is an immunosuppressive agent that has multiple targets such as cyclophilins and calcineurin [38]. Additionally, CsA only increases the threshold of _*m*_PTP inducers, so it would be desirable to explore another strategy to prevent the formation of _*m*_PTP and reperfusion injury. On the other hand, the use of Ru_360_ as a specific inhibitor of the MCU has been used in intact cardiac cells [7], isolated hearts [6, 10], and murine models [9] which provided protection against reperfusion injury, improving mechanical parameters, and decreasing infarct size and intracellular enzymes release [9, 39]. However, their pharmacokinetic parameters, bioavailability, and side effects have not been completely described.

Moreover, these therapeutic strategies that reduce ischemic injury could be beneficial to slow the progression of HF. In this regard, Santulli et al. [40] recently demonstrated that an intracellular Ca^2+^ leak could cause mitochondrial Ca^2+^ overload and dysfunction in HF murine models. In particular, a sarcoplasmic reticulum Ca^2+^ leak can establish a pathological feedback with mitochondria in which mitochondrial dysfunction increases ROS production, which consequently leads to oxidation/nitrosylation of some important sarcoplasmic reticulum channels such as RyR2, enhancing the diastolic Ca^2+^ leak and affecting cell contractility. Moreover, we found that selective MCU inhibition has the therapeutic potential to prevent catecholamine-induced toxicity as observed in HF [41]. In this regard, Anderson's group using MCU dominant-negative transgenic mice confirmed our findings of MCU's role on catecholamine response, because their mice were incapable of reaching catecholamine response [14, 27]. These novel data provide experimental rationale to explore the role of MCU during HF development.

At present, knockdown of gene expression by siRNA technologies offers a potent therapeutic approach to specific modulation of gene targeting [42]. siRNA therapeutics is suitable for drug use because it does not require genome integration and can be inexpensive and easily synthesized. Since a rational design of siRNA can specifically inhibit endogenous gene expression, it can modulate any disease-related gene expression [43]. Because of their great therapeutic potential, at least 20 siRNA-based drugs have now entered clinical trials in humans [43]. However, siRNA molecules are unstable in the serum and have shown poor cellular uptake and immunogenicity [44]. In this regard, the successful application of siRNA for therapy requires the development of effective drug delivery systems, in particular, nanovectors [45]. Recently, endothelial dysfunction present in HF has been explored as a novel avenue for the delivery of nanovectors, and the resulting endothelial permeability opens the field to nanotechnology-based therapies that could reach the myocardium through dysfunctional permeable endothelium [46]. In a murine model of HF, a passive intracardiac accumulation of high concentrations of nanocarriers occurred after a single application. Compared to the normal heart tissues, the accumulation of nanovectors was 12 times higher in HF animals [47]. This approach should be used as a potential avenue for siRNA-MCU therapy in reperfusion injury or HF, potentially translating towards novel therapies that might improve patient outcomes.

## 5. Conclusion

In summary, as the first proof of concept, we have shown that MCU silencing by specific siRNA reduces in vitro H/R injury as seen by cardiomyocyte death abatement. Therefore, this knowledge will allow for the development of a suitable delivery system to test the therapeutic potential of this siRNA in an in vivo model of mitochondrial calcium overload and dysfunction as those present in HF. In this context, the novel MCU druggability should be a fertile area of research in the future.

## Supplementary Material


**Supplementary Figure 1.** **A.** Characterization of MCU silenced cardiomyocytes. Viability of transfected cardiomyocytes measured with IP staining by flow cytometry. **B.** Comparison between forward scatter (FSC-A, size) and side scatter (SSC-A, complexity) between MCU-silenced cardiomyocytes and siRNA-Neg cells by flow cytometry. **C-D.** Representative traces and semiquantitative analysis of Δψm in MCU silenced and siRNA-Neg cardiomyocytes measured with 2 μM safranine in basal conditions, respectively. AFU, arbitrary fluorescence units; mean±SEM, *n* = 3. **E.** ATP levels in MCU silenced cardiomyocytes do not change respect to siRNA-Neg cardiomyocytes at basal conditions; mean ± SEM, *n* = 4. ATP measurement was performed with luminescent based luciferase assay. RLU, relative luminescence units.
**Supplementary Figure 2.** Mitochondrial permeability transition measurements in MCU-silenced cardiomyocytes.** A**. Representative mitochondrial Δψ traces in permeabilized MCU-silenced cardiomyocytes using 2 μM safranin, after the addition of 15 μM Ca^2+^. **B**. Representative Ca^2+^ retention traces in permeabilized transfected cardiomyocytes using CG-5N as a Ca^2+^ indicator after 50 μM Ca^2+^ addition. AFU, arbitrary fluorescence units. This experiment was performed in the presence or absence of 1 µM of CsA to inhibit _m_PTP opening. Traces are representative for at least 3 independent experiments.

## Abbreviations

AFU: Arbitrary fluorescence units
CG-5N: Calcium Green-5N
CCCP: Cyanide* m*-chlorophenyl hydrazone
CsA: Cyclosporin A
ETC: Electron transport chain
HR: Hypoxia/reoxygenation
MFI: Median fluorescence intensity
*m*Ca^2+^: Mitochondrial calcium
MCU: Mitochondrial calcium uniporter
_*m*_PTP: Mitochondrial permeability transition pore
Δ*ψ*_*m*_: Mitochondrial membrane potential
MVO_2_: Myocardial oxygen consumption
Nxy: Normoxy
PI: Propidium iodide
RLU: Relative luminescence units
ROS: Reactive oxygen species
SERCA: Sarco/endoplasmic reticulum calcium-ATPase
TG: Thapsigargin
TI: Tyrode ischemic solution
Uniplex: Mitochondrial calcium uniporter holocomplex.
