# Supplementary material for: Small Interfering RNA Targeting Mitochondrial Calcium Uniporter Improves Cardiomyocyte Cell Viability in Hypoxia/Reoxygenation Injury by Reducing Calcium Overload
Source: Oxid Med Cell Longev. 2017 Feb 27;2017:5750897. doi: 10.1155/2017/5750897 (PMC5350333; doi:10.1155/2017/5750897)
Supplement: Supplementary file 1 — Supplementary Figure 1. A. Characterization of MCU silenced cardiomyocytes. Viability of transfected cardiomyocytes measured with IP staining by flow cytometry. B. Comparison between forward scatter (FSC-A, size) and side scatter (SSC-A, complexity) between MCU-silenced cardiomyocytes and siRNA-Neg cells by flow cytometry. C-D. Representative traces and semiquantitative analysis of Δψm in MCU silenced and siRNA-Neg cardiomyocytes measured with 2 μM safranine in basal conditions, respectively. AFU, arbitrary fluorescence units; mean±SEM, n = 3. E. ATP levels in MCU silenced cardiomyocytes do not change respect to siRNA-Neg cardiomyocytes at basal conditions; mean ± SEM, n = 4. ATP measurement was performed with luminescent based luciferase assay. RLU, relative luminescence units. Supplementary Figure 2. Mitochondrial permeability transition measurements in MCU-silenced cardiomyocytes. A. Representative mitochondrial Δψ traces in permeabilized MCU-silenced cardiomyocytes using 2 μM safranin, after the addition of 15 μM Ca2+. B. Representative Ca2+ retention traces in permeabilized transfected cardiomyocytes using CG-5N as a Ca2+ indicator after 50 μM Ca2+ addition. AFU, arbitrary fluorescence units. This experiment was performed in the presence or absence of 1 µM of CsA to inhibit mPTP opening. Traces are representative for at least 3 independent experiments. [file 5750897.f1.docx]

**
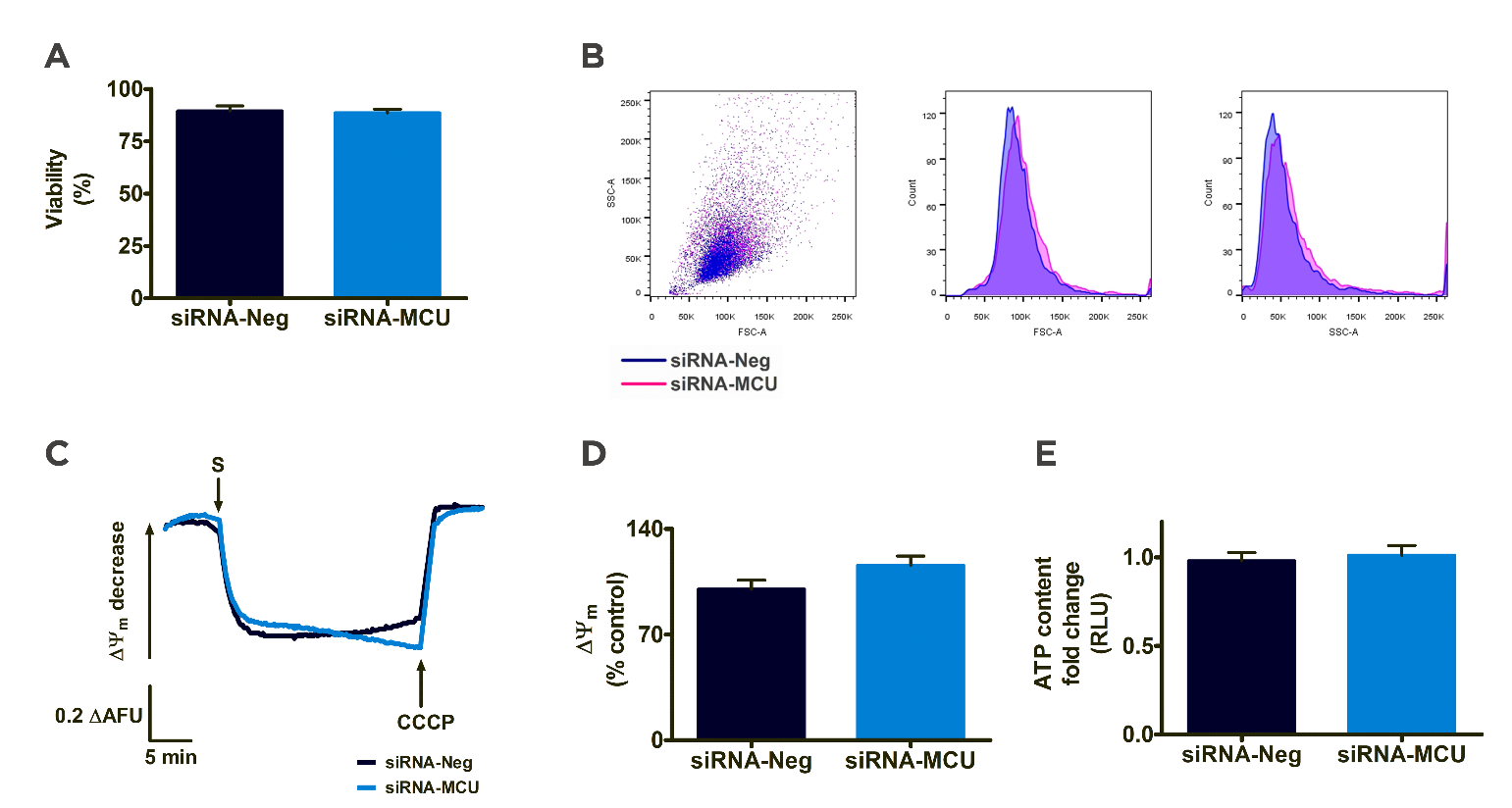
Supplementary Figure 1**

**Supplementary Figure 1. A.** Characterization of MCU silenced cardiomyocytes. Viability of transfected cardiomyocytes measured with IP staining by flow cytometry. **B.** Comparison between forward scatter (FSC-A, size) and side scatter (SSC-A, complexity) between MCU-silenced cardiomyocytes and siRNA-Neg cells by flow cytometry. **C-D.** Representative traces and semiquantitative analysis of Δψm in MCU silenced and siRNA-Neg cardiomyocytes measured with 2 μM safranine in basal conditions, respectively. AFU, arbitrary fluorescence units; mean±SEM, n=3. **E.** ATP levels in MCU silenced cardiomyocytes do not change respect to siRNA-Neg cardiomyocytes at basal conditions; mean±SEM, n=4. ATP measurement was performed with luminescent based luciferase assay. RLU, relative luminescence units.


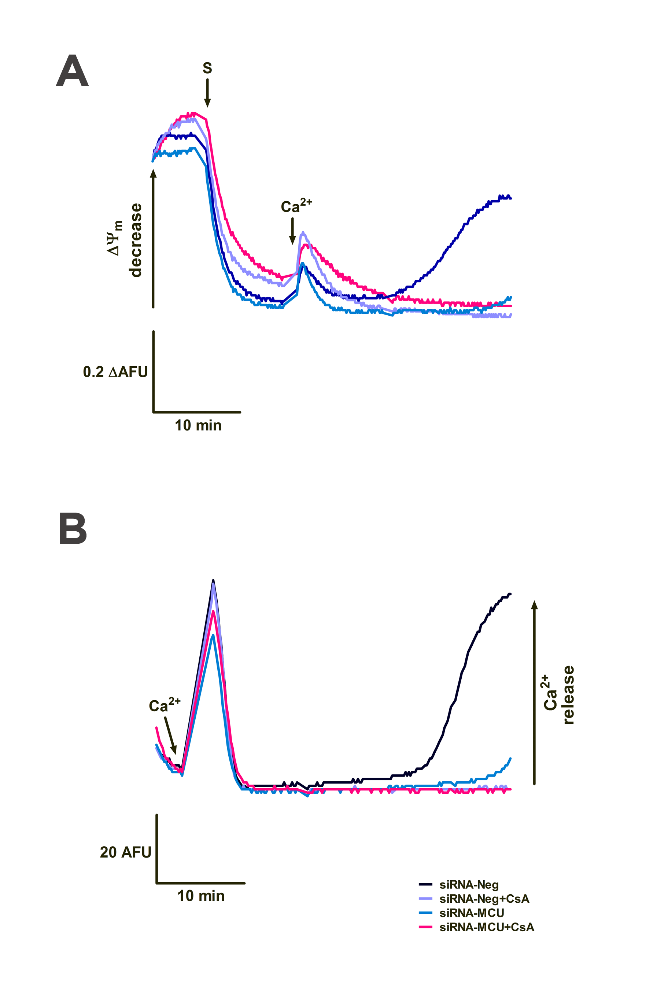
**Supplementary Figure 2**

**Supplementary Figure 2. Mitochondrial permeability transition measurements in MCU-silenced cardiomyocytes. A**. Representative mitochondrial Δψ traces in permeabilized MCU-silenced cardiomyocytes using 2 μM safranin, after the addition of 15 μM Ca^2+^. **B**. Representative Ca^2+^ retention traces in permeabilized transfected cardiomyocytes using CG-5N as a Ca^2+^ indicator after 50 μM Ca^2+^ addition. AFU, arbitrary fluorescence units. This experiment was performed in the presence or absence of 1 µM of CsA to inhibit _m_PTP opening. Traces are representative for at least 3 independent experiments.
